# Together again: the invasive mustard *Hesperis matronalis* suffers devastating seed predation by a recently adventive specialist weevil

**DOI:** 10.1007/s10526-025-10338-w

**Published:** 2025-08-05

**Authors:** David J. Ensing, Tyler D. Nelson, Chandra E. Moffat, Lauryn Joslin, Lucas Eckert, Marlene M. Kraml, Christopher G. Eckert

**Affiliations:** 1 Agriculture and Agri-Food Canada, Summerland Research and Development Centre, Summerland, BC Canada; 2 Department of Biology, Queen’s University, Kingston, ON Canada; 3 Department of Biology, McGill University, Montréal, QC Canada

**Keywords:** *Hesperis matronalis* (Brassicaceae: Hesperidae), *Ceutorhynchus inaffectatus* (Coleoptera: Curculionidae), Adventive, Fecundity, Invasive plant, Specialist

## Abstract

The enemy release hypothesis underpins classical (or importation) biocontrol as a management technique for invasive species. Classical biocontrol has had resounding success when prospective control agents have been subject to appropriate screening before release. Occasionally, however, natural enemies have been reunited with their hosts accidentally. Such adventive agents may provide effective control but have also avoided the careful screening characteristic of modern importation biocontrol programmes. We were studying the invasive mustard, *Hesperis matronalis* L. (Dame’s rocket; Brassicaceae: Hesperidae), when we discovered rampant seed predation by an unknown seed predator. Using DNA barcoding, we identified this seed predator as *Ceutorhynchus inaffectatus* Gyllenhal (Coleoptera: Curculionidae), a recently (2018) detected species in North America. Comparing potential and realised seed production, we found that seed predation by *C. inaffectatus* strongly reduces *H. matronalis* fecundity, and that this effect was not moderated by infection with turnip mosaic virus (TuMV), a commercially important pathogen hosted by *H. matronalis* and transmitted by polyphagous aphid species. *C. inaffectatus* is expected to be highly host-specific, and the absence of native Hesperidae species in North America suggests the potential for *C. inaffectatus* as a classical, but adventive, biocontrol agent of *H. matronalis*. We suggest population genetic research to identify the origin of *C. inaffectatus*, and host specificity testing before any intentional redistribution of this species for *H. matronalis* biocontrol. More generally, this system acts as a model for biocontrol prospects with adventive insect herbivore species.

**Supplementary Information:**

The online version contains supplementary material available at 10.1007/s10526-025-10338-w.

## Introduction

Management of introduced plants that go on to become invasive often involves reunification with specialist enemies from the pest’s native range (i.e., classical, or importation, biocontrol) in an attempt to reverse postulated enemy escape (enemy release hypothesis, reviewed by: Keane and Crawley 2002; Colautti et al. 2004; Brian and Catford 2023) and reduce both abundance and range expansion below economic and ecological thresholds. Contemporary biocontrol of invasive plants is subject to rigorous pre-release testing for host-specificity and efficacy, such that only highly specialist species with predicted efficacy are considered for release, often with spectacular success (Schwarzländer et al. 2018). For instance, St. John’s wort (*Hypericum perforatum*, Hypericaceae; Holloway and Huffaker 1951; Huffaker and Kennett 1959; DeLoach 1997), houndstongue (*Cynoglossum officinale*, Boraginaceae; Catton et al. 2016), and diffuse knapweed (*Centaurea diffusa*, Asteraceae; Myers et al. 2009) are all under successful control in North America as a result of self-sustaining invasive plant biocontrol programmes. Classical biocontrol by design selects only the most highly host specific and potentially efficacious natural enemies to be released –but what if a specialist enemy arrives on its own?

While studying introduced species, specialist enemies with unknown effects on the introduced species in their native range are occasionally –but with increasing frequency– being detected in the introduced range (Mason et al. 2017; Bertelsmeier et al. 2024; Müller-Schärer et al. 2024). Such ‘adventive’ natural enemies (Wheeler Jr. and Hoebeke 2017) may have arrived together (undetected) with the introduced pest of concern, migrated with unintentional human assistance, or been introduced surreptitiously, especially in regions without adequate regulation and/or enforcement (Weber et al. 2021). These adventive enemies may become invasive in their own right and may do so rapidly if suitable habitat including economically or ecologically important hosts are abundant. At the same time, if the enemy is sufficiently host-specific, it may prove a useful tool in controlling an invasive host (Müller-Schärer et al. 2014; Bonini et al. 2016; Augustinus et al. 2020; Müller-Schärer et al. 2024). Such specialist herbivores are expected to have limited potential to feed on native or economically important hosts and are therefore strong candidates as biocontrol agents.

*Hesperis matronalis* L. (Brassicaceae; dame’s rocket, dame’s violet, sweet rocket) is a biennial or winter annual that is native throughout Western Asia and Northwestern Europe (Hultén and Fries 1986) and was introduced intentionally to North America as garden ornamental in the 1700s (Mulligan 2002; Francis et al. 2009). It was considered naturalised by the mid 1800s in Québec, Canada, and is now found in 40 of 48 lower USA states, in all Canadian provinces and the Northwest Territories (https://plants.usda.gov/plant-profile/HEMA3; USDA PLANTS database, accessed 04 Dec. 2024) and considered invasive in several North American jurisdictions (Francis et al. 2009). *Hesperis matronalis* produces numerous flowers (mean = 75 per plant) that are visited by diverse insects, including bumblebees (*Bombus* spp. Latreille; Hymenoptera: Apidae), honeybees (*Apis mellifera* L.; Hymenoptera: Apidae), syrphid flies (Diptera: Syrphidae), sphinx moths (Lepidoptera: Sphingidae), and cabbage white butterflies (*Pieris rapae* L.; Lepidoptera: Pieridae; Majetic et al. 2007; Susko and Clubb 2008; Majetic et al. 2009). Flowers appear to be self-compatible though outcrossed flowers produce more seeds (Mitchell and Ankeny 2001; Susko and Clubb 2008; Francis et al. 2009). Each fruit contains 20–50 ovules (Susko and Clubb 2008), with observed mean seed set of 11 seeds per fruit (Mitchell and Ankeny 2001).

Like many Brassicaceae, *H. matronalis* is well defended by complex secondary metabolites, including several unique glucosinolates, that are recognised and used as cues by specialist and oligophagous herbivorous insects in the native range (Nair et al. 1976; Nielsen et al. 1989; Larsen et al. 1992; Montaut et al. 2020). However, in Canada, only the adventive *Delia radicum* L. (cabbage maggot; Diptera: Anthomyiidae) and *Plutella porrectella* L. (dame’s rocket moth; Lepidoptera: Plutellidae) had been reported feeding on *H. matronalis* with unknown influence on *H. matronalis* demography (Nair et al. 1976; Smith and Sears 1984; Francis et al. 2009). Seed predators were reported as absent before 2018 (Mitchell and Ankeny 2001; Pentinsaari et al. 2019). To our knowledge, no other herbivores have been reported feeding on *H. matronalis* in North America.

In its native range, *H. matronalis* supports a diverse community of phytophagous insects. In addition to *D. brassicae* and *P. porrectella*, *H. matronalis* herbivores include: the leaf feeding larvae of the Lepidopterans *Anthocharis cardamines* L. (Pieridae) and *Rhigognostis incarnatella* Steudel (Yponomeutidae), the specialist pollen beetle *Brassicogethes* (*Meligethes*) *matronalis* Audisio and Sporncraft (Coleoptera: Nitidulidae; Audisio et al. 2001) and its oligophagous congener *B. reitteri* Schilsky (Stevanovich and Audisio 1999). Neither *A. cardamines* nor *R. incarnatella* is known to occur in North America (GBIF and iNaturalist). Occurrence records are altogether absent for both *Brassicogethes* species in GBIF and iNaturalist, but they are not known to occur in North America either. Across Europe, *H. matronalis* seed pods are attacked by *Ceutorhynchus inaffectatus* Gyllenhall (Coleoptera: Curculionidae; Larsen et al. 1992; Korotyaev 2008; Francis et al. 2009), which was recently detected in North America (Pentinsaari et al. 2019). *Hesperis matronalis* is also occasionally infected by turnip mosaic virus (TuMV) in its native and introduced range (among other mosaic potyviruses), which is reliably diagnosed by colour breaking in the petals (Ford 1988; Francis et al. 2009; Lombardi et al. 2023). Mosaic potyviruses have been shown to alter host-plant interactions with other non-virus transmitting herbivores (Mauck et al. 2015), so infection of *H. matronalis* with TuMV may influence herbivory rates of adventive insect herbivores.

None of published literature on *H. matronalis* in North America mention seed predation. Yet, when we set out to assess its population demography in eastern Ontario, Canada, we found unexpected and severe feeding damage in the siliques (fruits). An unpublished thesis from 2006 does report seed damage, but it is not clear how proportional seed damage was calculated, and no seed predator was identified (Irazuzta 2006). Therefore, we sought to (1) identify the source(s) and origin of feeding damage we found in *H. matronalis* fruits, (2) evaluate whether this enemy (be it native or introduced) might act to control the spread of *H. matronalis*, and (3) investigate whether this novel damage interacts with potential damage caused by another non-native natural enemy, turnip mosaic virus.

## Materials and methods

To assess population demography of the target weed *H. matronalis*, in June 2021 we located 23 naturalized stands of *H. matronalis* over a 1300 km^2^ area within 40 km of Kingston, Ontario, Canada. We defined a stand as a discrete group of plants separated by at least 100 m from other such groups, and the median distance between stands was 500 m. In each stand we tagged 30–60 randomly chosen plants during peak flowering and diagnosed TuMV infection by colour breaking in the petals (Lombardi et al. 2023). In mid-August, when fruits were mature but seeds not yet released, we harvested the above-ground portion of all 243 plants that could be relocated with intact tags.

After drying plants to constant mass at 70 °C, we counted the number of mature fruits (each containing ≥ one mature, filled seed) plus the number of flowers that failed to develop fruits (indicated by persistent petioles). We estimated above-ground size by total dry stem mass, because about 30% of plants had some of their leaves at this stage. We estimated flower number as the sum of fruit number and the number of fruits that did not develop. We randomly selected five fruits from each plant, counted the number of mature, filled seeds in each and estimated total lifetime seed production for each plant as the product of fruit number and the average number of filled, undamaged seeds per fruit. We unexpectedly found that many seeds were destroyed by a pre-dispersal seed predator, so to estimate the number of seeds that could have potentially matured in the absence of seed predation, we counted the indentations left by developing seeds in the silique septum. Only ovules that develop into a size approaching a mature seed will leave a distinct indent in the fruit septum. Accordingly, the total number of indents in a fruit indicates the number of mature seed plus seeds lost to predation plus seeds that were aborted at a late stage. Ovules that were aborted at a late stage were extremely rare in the fruits we analyzed. This agrees with results from Susko and Clubb (2008) who found that only 3% of ovules were aborted at late stage. This supports our assumption that the loss of developing ovules large enough to leave indents in the septum was overwhelmingly caused by seed predation. From these data, we estimated the proportion of seeds destroyed and potential lifetime seed production in the absence of seed predation. TuMV infection can reduce *H. matronalis* size (36.6% smaller) and seed production (52% fewer seeds; Joslin 2022), but it was unknown if seed predation exacerbated the negative effect of TuMV on *H. matronalis*. We therefore compared seed predation between infected and uninfected plants to assess a possible interaction between TuMV and the seed predator.

### Statistical analysis of fitness components

We performed all statistical analyses using the R statistical environment version 4.4.1 (R Core Team 2024). In general, individual-level seed production correlates strongly with size in plants. To evaluate whether seed predation affected the correlation between measures of plant size and lifetime seed production, we fit either potential seeds/plant (in the absence of seed predation, see above) or realized seeds/plant (in the presence of seed predation) to a generalized linear mixed-effects model with the measure of plant size (dry stem mass, flower number or fruit number) as a fixed predictor and stand as a random effect using the glmmTMB function in the glmmTMB R package (version 1.1.9, Brooks et al. 2017). All predictors and response variables were lognormally distributed hence were log_10_-transformed for analysis. Predictor and response variables were standardized so that the regression coefficients were equivalent to correlation coefficients for comparison between analyses. We evaluated whether measures of plants size predicted the proportion of fruits damaged or seeds destroyed using glmmTMB with plant size as predictor and seed predator damage as response (Binomial errors, logit link function). For both sets of analyses, significance of predictors was evaluated using χ^2^ likelihood ratio tests performed using the anova function in R.

To determine whether fruit or seed damage by the seed predator differed between plants infected or uninfected with TuMV, we used glmmTMB to fit the proportion of fruits incurring some seed predation and the proportion of seeds destroyed as binomial response variables to generalized linear mixed models with infection status as a fixed predictor and stand as a random effect (logit link function). Significance was evaluated using likelihood ratio tests as above.

### DNA barcoding of unknown larval seed predator

In the fruit samples analyzed above, we found 35 live seed-predating insect larvae from 11 of 22 stands (Supplementary Table S1), which we identified as beetles (Coleoptera). We preserved them individually in 95% ethanol. All were morphologically similar and we used DNA barcoding to genotype eleven (representing 7/11 stands with live larvae). We extracted genomic DNA from each larva using a DNeasy Blood & Tissue Kit (QIAGEN, Hilden, Germany) following the manufacturer’s protocol with the addition of RNase A (4 ul at 100 mg ml^-1^; QIAGEN). To increase DNA concentration, we eluted DNA into two aliquots of 50 ul Buffer AE heated to 56 °C. We used PuReTaq Ready-To-Go PCR Beads (GE Healthcare, Chicago, Illinois, USA) and the ‘universal’ primer set (Folmer et al. 1994; Integrated DNA Technologies, Coralville, Iowa, USA) to amplify the barcode region of the mitochondrial cytochrome *c* oxidase subunit I (COI) gene by following the PCR protocol of Gariepy et al. (2014). We purified amplicons with ExoSAP-IT Express (Applied Biosystems/Thermo-Fisher Scientific) following the manufacturer’s protocol. Bidirectional Sanger sequencing was completed at the University of British Columbia, Canada with BigDye version 3.1 on an Applied Biosystems 3730S DNA Analyzer. We generated and edited consensus COI barcode sequences using ApE version 2.0.61 (Davis and Jorgensen 2022), then used MegaBLAST (default module of BLAST + version 2.13.0), to search the National Center for Biotechnology Information archive for close nucleotide matches (Madden 2002). As all specimens were preserved as larvae, we were unable to morphologically verify the identify beyond insect order. BOLD sample accessions, under project “HMCI”, are included in Supplementary Table S2.

### Phylogenetic analysis

The COI sequences of all our insect specimens were consistent with other COI sequences of *Ceutorhynchus inaffectatus*, of which several have expert taxonomic identifications based on morphology (see Results). We thus further compiled all *C. inaffectatus* COI sequences from the Barcode of Life Database (BOLD; Ratnasingham and Hebert 2007), and included up to five BOLD records of other *Ceutorhynchus* Germar species, including: *C. alliariae* Brisout De Barneville, *C. arator* Gyllenhal, 1837; *C. peyerimhoffi* Hustache, and *C. roberti* Gyllenhal, 1837 (Supplementary Table S2). For the outgroup, we included one *Mogulones* sequence, a member of the same tribe (Ceutorhynchini) as *Ceutorhynchus* (Letsch et al. 2024). We aligned all sequences in AliView version 1.28 (Larsson 2014) using MUSCLE version 3.8.425 (Edgar 2004) then trimmed the resulting multiple sequence alignment. We built a maximum likelihood gene tree using IQ-TREE version 1.6.12 (Nguyen et al. 2015) using ModelFinder (Kalyaanamoorthy et al. 2017) to identify the phylogenetic model with greatest support across 1000 ultrafast bootstraps (Hoang et al. 2018). ModelFinder determined that the K3Pu + F + G4 substitution model was most suitable for analysis of our 521 base-pair multiple sequence alignment of 53 total sequences. We visualized this tree in FigTree version 1.4.4 (Rambaut 2018).

## Results

### Seed predator identification

**Fig. 1:**
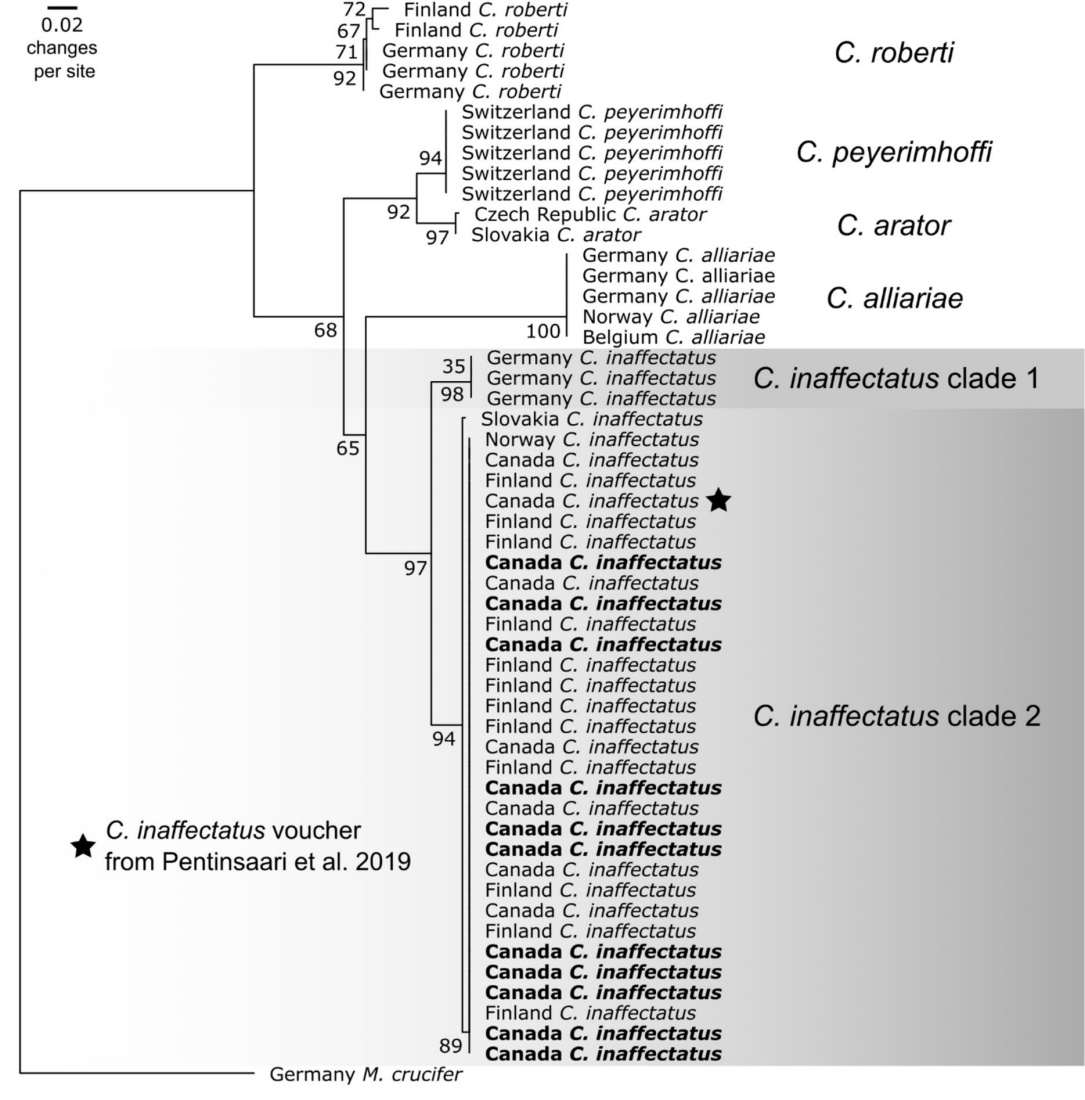
Maximum likelihood gene tree of our 11 unknown samples (in bold face), and 24 *Ceutorhynchus inaffectatus*, five *C. alliariae*, two *C. arator*, five *C. peyerimhoffi*, and five *C. roberti* cytochrome *c* oxidase subunit I DNA barcodes from the Barcode of Life Database, indicating a 100% match between our samples and existing public *C. inaffectatus* sequences from Ontario, Canada, Norway, and Finland. Node values indicate bootstrap support. Scale bar depicts average number of nucleotide substitutions per site. Grey areas separate *C. inaffectatus* clades 1 (German samples) and 2 (all other samples, including ours) from other *Ceutorhynchus* species

DNA barcoding and subsequent comparison against the National Center for Biotechnology Information archive indicated that all 11 samples were 100% matches with 21 of 24 public *C. inaffectatus* BOLD sequences (Coleoptera: Curculionidae; clade 2, Fig. 1). Among these matches was the *C. inaffectatus* voucher specimen that confirmed the species was adventive in Canada (Pentinsaari et al. 2019). Maximum likelihood analysis revealed that three *C. inaffectatus* DNA barcode samples from Germany differed by about 4% from the samples from Ontario, Canada, Slovakia, Norway, and Finland (clade 1, Fig. 1).

### Fitness consequences

The plants we sampled varied widely in size and lifetime reproductive success. Dry stem mass varied 100-fold among individuals (0.40–72.65 g) with a median of 5.47 g. The number of flowers and fruits produced per individual varied more than 100-fold (5–736 flowers, median = 62; 5–722 fruits, median = 95). The number of filled seeds per plant varied even more dramatically (0–10 146, median = 323). Seed predation was extensive, with 98.8% of 243 plants sampled having at least one fruit damaged by the seed predator and 72.0% suffering damage to all five fruits sampled. On average, 75.7% of seed was eaten by the seed predator (range = 36.4–100.0%, median = 76%) and 3.6% had all seed destroyed.

**Fig. 2:**
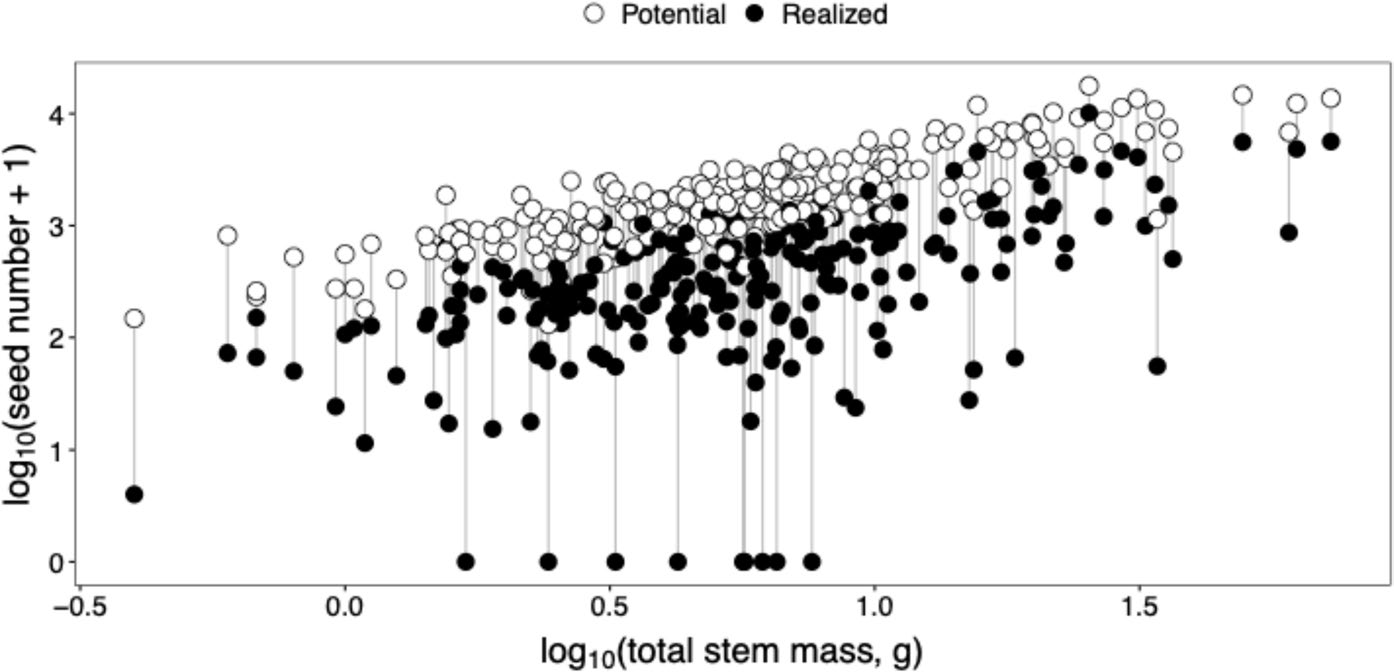
Seed predation weakens the relationship between plant size and realized seed production. Each pair of points is an individual *Hesperis matronalis* (*n* = 243) with the open symbol representing potential seed production (without predation) and the closed symbol realized seed production (with predation). The length of the grey line joining the two points in a pair shows the proportional loss of seed production (in log_10_ seed number) caused by predation. Analysis of these data is presented in Table 1

Seed predation weakened the correlation between plant size and seed production (Fig. 2; Table 1). Standardized regression coefficients (i.e., correlation coefficients) from a mixed-effects regression of potential seed production (range = 130–17728, mean = 2511.5, median = 1591.2) over a measure of plant size (dry stem mass, flower number, or fruit number) were very strong (*r* = 0.833–0.956), whereas those from realized seed production (0–10146, median = 323, mean = 683.6) were much weaker (*r* =  0.450–0.563). Measures of plant size correlated weakly but positively with the proportion of fruits incurring some damage but did not correlate with the proportion of seeds damaged (Table 1).

**Table 1:** Seed predation weakens the correlation between plant size and seed production (compare *r* for potential seeds/plant to realized seeds/plant) but fruit and seed damage is not well predicted by measures of plant size

	Standardized regression coefficient (*r*, and its SE)			
Measure of plant size	Potential seeds/plant	Realized seeds/plant	Proportion of fruits damaged	Proportion of seeds destroyed
log_10_(dry stem mass)	+ 0.833 (0.034), *P* < 0.0001	+ 0.450 (0.049), *P* < 0.0001	+ 0.145 (0.062), *P* = 0.018	–0.024 (0.054), *P* = 0.65
log_10_(flower number)	+ 0.933 (0.025), *P* < 0.0001	+ 0.533 (0.047), *P* < 0.0001	+ 0.139 (0.064), *P* = 0.029	–0.058 (0.055), *P* = 0.29
log_10_(fruit number)	+ 0.956 (0.022), *P* < 0.0001	+ 0.563 (0.047), *P* < 0.0001	+ 0.125 (0.064), *P* = 0.051	–0.073 (0.056), *P* = 0.19

Cells contain standardized regression coefficients (akin to correlation coefficients) from mixed-effects linear models with a measure of plant size as a single fixed predictor (df = 1) and stand as a random effect. *P* values are from χ^2^likelihood ratio tests

Overall, 11.5% of sampled plants were infected with TuMV. The proportion of fruits incurring seed damage by the seed predator was 2.6% lower among infected plants but the proportion of seeds destroyed was 6.9% higher, though both differences were not quite significant (Table 2).

**Table 2:** Comparison of the proportion of fruits damaged and seeds destroyed by the seed predator between plants infected and uninfected with turnip mosaic virus

	Proportion of fruits damaged	Proportion of seed destroyed
Uninfected plants (*n* = 215)	0.889 ± 0.015	0.751 ± 0.010
Infected plants (*n* = 28)	0.866 ± 0.044	0.803 ± 0.029
Mixed model comparison	χ^2^ = 3.15, *P* = 0.076	χ^2^ = 3.72, *P* = 0.054

Cells contain means ± SE. Infected plants were compared with uninfected plants using a generalized linear mixed model with infection status as a single fixed effect (df = 1) and site as a random effect. The significance of infection was evaluated with a χ^2^ likelihood ratio test

## Discussion

### Seed predation by *C. inaffectatus* dramatically reduces *H. matronalis* fitness

Introduced and naturalized *H. matronalis* in eastern Ontario, Canada suffers severe loss of fitness due to seed predation by an adventive natural enemy, *C. inaffectatus*. Nearly 99% of plants sampled (n = 243) lost seeds to the weevil, while more than 70% had seed predation in all sampled fruits, indicating substantial pressure from *C. inaffectatus*. Potential fecundity of *H. matronalis* correlated strongly with individual plant size (*r* > 0.83), a pattern common among plants (Aarssen and Taylor 1992) but especially so in short lived species (i.e., annuals, or biennials like *H. matronalis*; e.g., Aarssen and Jordan 2001), where the resource rich individuals are larger and more fecund. However this relation was almost cut in half by seed predation, with actual seed production being only weakly related to plant size (*r* < 0.56; Table 1). The eventual fitness cost (i.e., proportion seeds predated) did not correlate with plant size, indicating that gravid *C. inaffectatus* females do not select hosts based on their final size.

### *C. inaffectatus* in North America

Here, we report for the first time the strong influence of *C. inaffectatus* on *H. matronalis* fecundity in North America, with our 2022 field season documenting widespread feeding on populations near Kingston, Ontario, Canada. *C. inaffectatus* was first reported in North America in Guelph, Ontario in 2018 (Pentinsaari et al. 2019), and, despite limited iNaturalist detections globally (< 30), *C. inaffectatus* was reported in iNaturalist as early as 2020 near Toronto and Vaughan, Ontario, and in Albion, Michigan, USA (https://www.inaturalist.org/observations?subview=table&taxon_id=496827, accessed 04 Dec 2024). By 2024, ‘research grade’ North American iNaturalist records included more occurrences around Toronto, Canada and Michigan, as well as in Ithaca, New York, USA. In nearly all cases, observers report strong associations and high abundance on *H. matronalis* (https://www.inaturalist.org/observations?subview=table&taxon_id=496827; accessed 04 Dec 2024). The Global Biodiversity Information Facility (https://www.gbif.org/) includes many more records in Western and particularly Northwestern Europe (Nielsen et al. 1989; Larsen et al. 1992; Nielsen et al. 1995), but does not add North American occurrences beyond the iNaturalist records above.

### Interactions with pathogens

*Hesperis matronalis* in our study area were often infected with turnip mosaic virus (TuMV; > 10% of plants), and this infection appears to reduce *H. matronalis* size and potential fecundity, although experimental infections are required to confirm a direct effect. However, TuMV infection did not appear to interact with *C. inaffectatus* predation in influencing *H. matronalis* fitness. TuMV infection varied among populations (Dawson 2023), and some populations may even harbour resistance to infection (Lombardi et al. 2023). Turnip mosaic virus is a widespread and problematic pathogen of Brassicaceae species, including other wild (wild *Brassica oleracea*; Maskell et al. 1999) and important cultivated species, with the potential to cause significant yield losses (e.g., Spence et al. 2007). Moreover, TuMV is spread by at least 40 species of aphid, and has evolved resistance-breaking strains against *B. napus* (oilseed; Guerret et al. 2017). The potential for *H. matronalis* to act as a TuMV reservoir and potentially a stepping stone species (Papaïx et al. 2015) in both space and time has potential consequences for cultivated brassicas in North America, and should prioritise *H. matronalis* for control.

### Risk to native species from *C. inaffectatus*

Our findings indicate the potential for effective biocontrol of *H. matronalis* by *C. inaffectatus*, but could this species pose a risk to native species, especially other Brassicaceae? The risk in North America is likely low. Like many mustards, *H. matronalis* is well defended by specific combinations of glucosinolates (Montaut et al. 2020), and *C. inaffectatus* is sensitive to these (Larsen et al. 1992). As a result, it is likely that *C. inaffectatus* is specific to the genus *Hesperis* (Dieckmann 1972), with some considering it specific to *H. matronalis* (Larsen et al. 1992; Pentinsaari et al. 2019). Dieckmann (1972) reports that *C. inaffectatus* was found on *H. tristis* in Germany and Austria. However, we found substantial barcode divergence (> 4%) between the German *C. inaffectatus* samples and those from Canada, the Fennoscandian Peninsula, and Slovakia (Fig. 1), thus it is possible the German populations currently called *C. inaffectatus* represent an as-of-yet undescribed cryptic species. Detailed information on the host plants from which the samples were collected would clarify any potential host-associated barcode divergence, and we encourage further systematic study of both *C. inaffectatus* and *H. matronalis*, and their closely related congeners, in their native range. Finally, we also found one report (Yunakov et al. 2018) suggesting *C. inaffectatus* presence on field mustard (*Rhamphospermum* (*Sinapis*) *arvensis*), but this is not confirmed with any other reports in Turkey (Gültekin and Korotyaev 2001), Western or Northern Europe (Larsen et al. 1992), or North America (Pentinsaari et al. 2019; https://www.inaturalist.org/records).

The potential for *C. inaffectatus* also seems high because the Brassicaceae tribe Hesperideae is monogeneric, including only *Hesperis*, and none of the 25 species of *Hesperis* is native to North America (Al-Shehbaz et al. 2006; Eslami-Farouji et al. 2021). Only *H. matronalis* has been introduced. Species in the most closely related Brassicaceae tribes are rare in North America: Euclidieae (17 *Braya* spp. of 150 total species), Anchonieae (4 *Parrya* spp. of ~ 130 total species), and Chorisporeae (12 Asian species) (Al-Shehbaz et al. 2006). However, since many Brassicaceae are economically significant (e.g., *Brassica oleracea*, *B. rapa*, *Sinapis* spp., *Raphanus* spp.; all tribe Brassiceae) and despite the relatively distant relatedness between tribes Brassiceae and Hesperideae (Al-Shehbaz et al. 2006; Eslami-Farouji et al. 2021), host specificity testing of *C. inaffectatus* is warranted prior to any potential redistribution efforts. Nevertheless, *Ceutorhynchus* species are generally specific to Brassicaceae (but some also feed on Resedaceae and Capparaceae, both Brassicales; Korotyaev 2008; Letsch et al. 2018), so much so that *Ceutorhynchus* specimens preserved in amber have been used to try to date the origins of Brassicaceae (e.g., Legalov et al. 2022). *Ceutorhynchus* species are also often monophagous, or at most oligophagous. Emphasizing their tendency for host-specificity, some *Ceutorhynchus* species are already released or under consideration for biocontrol of invasive Brassicaceae species in Canada, like garlic mustard (*Alliaria petiolata*: *C. scrobicollis* (released), *C. constrictus* (petitioned); McTavish et al. 2024) and hoary cresses (*Lepidium draba, L. chalapense*, and *L. appelianum: C. carderiae, C. turbatus*; Weyl et al. 2024). Given the likelihood of host specificity and substantial seed predation we report *C. inaffectatus* is a strong candidate for biocontrol of *H. matronalis* in North America.

### *Biocontrol of* Hesperis matronalis*?*

The enemy release hypothesis underpins importation biocontrol programmes, and in this context depends on three key themes: (1) the specialist enemies of the target species will be absent from the adventive region, (2) specialist enemies of native congeners do not switch to the target, and (3) native generalist herbivores will damage native competitors more than the target (Keane and Crawley 2002). It is logical that an invasive plant-biocontrol agent system that satisfies these requirements stands the best chance at successful biocontrol, and indeed these are well met by *H. matronalis*-*C. inaffectatus* system throughout North America. First, *H. matronalis* has been present in North America likely since the 1700s, and naturalised since at least the middle nineteenth century (Francis et al. 2009), in the absence of its specialist herbivores. *C. inaffectatus* has only been detected in North America since 2018 (Pentinsaari et al. 2019), such that it is likely there have been 300 years during which *H. matronalis* could evolve in the absence of this seed predator. Second, there are no native congeners of *H. matronalis* in North America (Al-Shehbaz et al. 2006; Eslami-Farouji et al. 2021), so host-switching by native specialists on congeners is not possible. And third, and perhaps most pertinently, *H. matronalis* is well defended chemically (Nair et al. 1976; Larsen et al. 1992; Montaut et al. 2020), and appears to receive little, if any, damage from native generalists in North America. In Canada, only the adventive *D. brassicae* (cabbage maggot) and *P. porrectella* (dame’s rocket moth) were reported feeding on *H. matrionalis* (Nair et al. 1976; Smith and Sears 1984; Francis et al. 2009), before *C. inaffectatus* was detected in 2018, although we did not detect them or anything else feeding on *H. matronalis* in our study sites. Since it meets these criteria, and given the specificity outlined above, *C. inaffectatus* is a strong candidate for biocontrol of *H. matronalis* (Pentinsaari et al. 2019) and is reducing *H. matronalis* fecundity when present (Fig. 2).

Adventive insects can be effective biocontrol agents, but they will have lacked the rigorous pre-release testing required by intentional releases (Weber et al. 2021). Provided they prove to be host specific, they may even be more effective, as they have avoided ‘domestication syndrome’ that results from rearing in quarantine facilities common in modern importation biocontrol, and which can result in inbreeding depression and associated negative effects on fitness (Woodworth et al. 2002; Szűcs et al. 2019). Alternatively, they may have suffered the strong founder effects that often characterise unintentionally introduced species (Eckert et al. 1996; Hagan et al. 2024). Applying population genomic tools to compare North American to European populations of *C. inaffectatus* would help reveal its origins (and potential pathways of introduction). Moreover, if *C. inaffectatus* is suffering from reduced genomic variation as a result of founder effects, its efficacy and/or potential spread in North America may be limited and a biocontrol program would benefit from sourcing additional genotypes. Population genomic data would thus inform potential collection activities in the native range to mitigate founder effects of an intentional, importation biocontrol program using *C. inaffectatus* for *H. matronalis* in North America.

### Adventive introductions – a biosecurity risk

Insects are continually colonizing outside their native range, and most of the extreme range expansions are facilitated by human activity. Among the insects that successfully colonise, some like *C. inaffectatus* just happen to be a specialist on a species that we consider invasive (Weber et al. 2021). For instance, like *H. matronalis* and *C. inaffectatus* in North America, common ragweed invasive in Northern Italy is experiencing effective control by the adventive North American native beetle *Ophraella communa* (Coleoptera: Chrysomelidae; Müller-Schärer et al. 2014), which has reduced pollen concentrations by 80%, leading to substantial healthcare savings (Bonini et al. 2016). Other adventive insect introductions, in fact probably most, will have broader host preferences and may therefore damage native and/or economically important species. In other words, the introduction of *C. inaffectatus* to North America is part of a larger biological phenomenon that reflects the increasing connectivity of humans globally. Community science initiatives (e.g., Bioblitzes) and applications (e.g., iNaturalist) will continue to improve our understanding of species introductions, and our capacity to respond to the biosecurity risk adventive species represent (Koen and Newton 2021; Dimson et al. 2023; Potgieter et al. 2024).

## Supplementary Information

Below is the link to the electronic supplementary material.

Supplementary file1 (CSV 2 KB)

Supplementary file2 (CSV 3 KB)

## Data Availability

Raw data and analysis code are available from the authors on request.

## Declarations

### Research involving human and animal participants

Our research did not involve human participants or animals, and did not require informed consent.
